# Cross-sectional study on user requirements for developing a digital patient navigator app

**DOI:** 10.1177/20552076251387746

**Published:** 2025-10-28

**Authors:** Katharina Seitz, Manuela Langbein, Chloë Goossens, Stefanie Altmannshofer, Peter A. Fasching, Carolin C. Hack, Felix Heindl, Matthias W. Beckmann, Julius Emons, Hanna Huebner

**Affiliations:** 1 Department of Gynecology and Obstetrics, 27168 Universitätsklinikum Erlangen, Friedrich-Alexander-Universität Erlangen-Nürnberg (FAU), Erlangen, Germany; 2 Comprehensive Cancer Center Erlangen-EMN (CCC-ER-EMN), Erlangen, Germany; 3 Bavarian Cancer Research Center (BZKF), Erlangen, Germany

**Keywords:** mHealth, patient navigator, digital health, breast cancer, mobile apps

## Abstract

**Objective:**

This study aimed to assess patients’ preferences regarding the content and features of a digital patient navigator app. A secondary objective was to explore how patient characteristics influence the perceived importance and anticipated frequency of use of different app components.

**Methods:**

The study was a monocentric cross-sectional survey conducted at the University Hospital Erlangen. A questionnaire consisting of 20 questions was designed to inquire about patients’ preferences for a digital patient navigator. Participants had breast diseases. Descriptive analyses were conducted to identify key app features and their correlation with patient characteristics.

**Results:**

Questionnaires from 243 patients were analyzed. Preferred key app features, in order of preference from highest to lowest, were as follows: a communication feature to chat with treating physicians, a personalized treatment plan, real-time notifications, mediation services for providing support, 3D navigation within the hospital, and personal documents. Patients’ age, native language, and education significantly influenced the assessment of importance and frequency of use of some app features. A personalized treatment plan was considered more important by younger individuals (≤60 years, *p* < 0.001), whereas real-time notifications were considered more important by participants with a lower educational level (*p* = 0.003) and younger individuals (*p* = 0.036). Increased frequency of use of a personalized treatment plan tool was also associated with younger age (*p* < 0.001) and lower education levels (*p* = 0.025).

**Conclusion:**

These findings suggest that a patient navigator app could be a valuable tool for a broad range of patients, potentially complementing in-person patient navigators. To ensure broad usability and acceptance, future development should account for varying needs across patient subgroups—particularly in terms of personalization, language accessibility, and communication features.

## Introduction

Patient navigators play an important role in various aspects of medical care, acting as a bridge between patients and healthcare professionals. Different models of patient guidance have been developed internationally and, more recently, in Germany.^1–4^ Such roles are now increasingly integrated into large hospitals to offer guidance and support to patients and their families. The assistance provided by patient navigators ranges from offering essential information and highlighting existing services to providing practical support, such as accompanying patients to appointments, organizing appointments, or assisting with formalities.^2,5,6^ These pilot projects—likely initiated due to the shortage of physicians—demonstrate that a long-term shift toward a new division of responsibilities in the healthcare system has already begun. This shift involves a multiprofessional team and greater patient involvement in decision-making. The delegation of services also promotes the professionalization of nonphysician staff.^7^ The introduction of in-person patient navigators offers numerous advantages, and various studies have shown that they can reduce hospitalizations and improve both patient health and perceived quality of care.^8–11^ While direct, on-site care provided by in-person patient navigators remains indispensable in many cases, certain forms of assistance and guidance could be supplemented—or even fully managed—by a digital navigator app. The high number of patients makes it impossible to provide individual guidance to each one. In 2022, nearly 70,000 patients received inpatient or day-care treatment at the University Hospital Erlangen, including 8500 patients at the Department of Gynecology and Obstetrics. Additional support for the existing patient navigators is therefore urgently needed, and a digital solution may be the most viable option.

Many apps support our daily lives, and electronic (eHealth) and mobile health (mHealth) applications are a rapidly growing trend in the medical field. Interest in electronic access to health information continues to increase due to its convenience, accessibility, and efficiency.^12–15^ Especially for patients with chronic diseases or cancer, apps can improve healthcare and disease management.^16^ They are also effective tools for enhancing quality of life, extending survival^17–19^ and positively influencing individual health behaviors.^20^ Prior research has shown that cancer patients are willing to use digital health technologies if developed through a patient-driven approach,^21^ making them valuable tools for self-management.^22^

This paper presents the first steps toward developing a digital patient navigator that combines the benefits of digitalization with user-friendly guidance tools. Such a health application could offer advantages for both patients and clinicians. For patients, benefits would include an optimized hospital experience, access to up-to-date information about their therapy, and a more personalized, patient-centered support system—potentially improving satisfaction and treatment quality. This may ultimately lead to better health outcomes. For clinicians, it could reduce workload and save time during medical appointments.

The overall aim of this study was to actively involve patients in the design and development of a digital patient navigator app, ensuring their needs and preferences were fully considered. Engaging patients early is essential to facilitate the development of patient-centered apps with content that is driven by the lived experiences of end users.^23,24^ Codesign strategies have been shown to improve the chance of a successful eHealth development and the seamless implementation into practice.^25,26^ While the inclusion of healthcare professionals is known to enhance the quality of digital health solutions, early and meaningful involvement of patients is equally important.^27,28^ Accordingly, the specific objectives of the study were as follows: (1) to identify and describe potential key features of the app and to assess their importance and anticipated frequency of use and (2) to evaluate potential variations in expectations regarding importance and frequency of use among different patient groups. The insights gained from this study are intended to guide the development of a prototype.

## Materials and methods

### Study design and population

This study was conducted as an anonymous cross-sectional survey between September 2023 and November 2023. Patients were recruited at the Department of Gynecology and Obstetrics at the University Hospital Erlangen, either during waiting periods or consultation hours at the breast clinic, preoperative clinic, or oncology day clinic. The patient population can, therefore, be divided into three main groups: patients with malignant breast disease, patients with benign breast disease, or patients with a breast condition of unclear diagnosis.

Participants were eligible if they met the following inclusion criteria: (1) aged 18 years or older and (2) diagnosed with breast cancer, a benign breast disease, or unclear breast findings. Exclusion criteria were as follows: (1) inability to understand the German language, making it impossible to complete the questionnaire, and (2) difficulty understanding the content of the study or questionnaire.

All patients visiting the breast clinic, preoperative clinic, or oncology day clinic were informed about the study verbally by study staff and, in case of interest in the study and sufficient time to fill in the questionnaire received a short, written explanation outlining the purpose of the survey and the voluntary, anonymous nature of participation. No incentives were offered. While the total number of distributed questionnaires was not systematically recorded, 259 questionnaires were returned. Due to this limitation, an exact response rate could not be calculated. Eleven responses were excluded due to unrelated diagnoses, and five were excluded because the questionnaire could not be completed independently, resulting in a final study population of 243 individuals. As the survey was fully anonymous and involved no identifying information or clinical interventions, no written informed consent was required. The study was conducted in accordance with the Declaration of Helsinki and approved by the Ethics Committee of the Medical Faculty, Friedrich-Alexander University Erlangen-Nürnberg (protocol code 23-327-ANF).

### Questionnaire design

The questionnaire was newly developed for this study. Its structure and items were informed by a literature review on patient navigation needs, mHealth tools in oncology, and digital health engagement. In addition, three experienced in-person patient navigators from the Comprehensive Cancer Center Erlangen-EMN were consulted in advance and interviewed regarding their observations and expectations about patients’ needs. The insights obtained from those interviews were incorporated into the questionnaire—particularly in the section “expectations of a digital patient navigator.” The final items were developed by an interdisciplinary team of clinicians, researchers, and the patient navigators. To support the development of a digital navigator app, it is important to consider patients’ previous experiences with in-person patient navigators and the types of support already provided. Thus, a question assessing the previous contact with in-person patient navigators was added.

A draft version of the questionnaire was pretested with five previously noninvolved volunteers to evaluate clarity and usability. Minor revisions were made based on their feedback. The final questionnaire consisted of 20 questions, categorized into the following sections: (1) demographic information, (2) reason for presentation, health status, and experience with the healthcare system, (3) experience with patient navigators, and (4) expectations of a digital patient navigator. The questions were formatted as multiple-choice, polar (yes/no), free-text, or Likert scale-based. Questions assessing the importance and frequency of use of different app features were scaled using a 5-point Likert scale, ranging from “very important” (5 points) to “not important at all” (1 point), or analogously, from “very often” (5 points) to “very rarely” (1 point). The app features evaluated included: (1) digital 3D guidance, (2) infotexts and videos, (3) patient community, (4) access to resources and self-help materials, (5) contact with in-person patient navigators, (6) digital information and mediation activities, (7) personalized treatment plan, (8) real-time notifications, (9) direct communication with attending physicians, and (10) anonymous feedback. A translated version of the German questionnaire is provided in Supplementary File 1. Based on the answers given, groups were defined as follows for comparison: (1) age (≤60 years vs. >60 years), based on evidence that digital health usage tends to decline in individuals over 60 years of age,^29^ (2) first language (German vs. other languages), (3) educational status (<high school diploma vs. ≥high school diploma), (4) previous clinic experience (yes vs. no), and (5) previous contact with in-person patient navigators (yes vs. no).

### Data analysis

Statistical analysis was performed using Microsoft Excel (Version 2019) for descriptive statistics (numbers and frequencies), IBM SPSS Statistics (Version 29.0.1.0) for inferential statistics, and GraphPad Prism (Version 9.5.1) for data visualization. Continuous variables were presented as mean ± standard deviation, and categorical variables as absolute and relative frequencies. Associations between patient characteristics and the perceived importance and frequency of use of different app features were evaluated using the Mann–Whitney *U* test, with a significance level set at *p* < 0.05. The Mann–Whitney *U* test was chosen due the variables analyzed not following a normal distribution (Likert scale). Given the exploratory nature of the study, no correction of multiple testing was performed to avoid missing relevant associations. Reported *p*-values should therefore be interpreted with caution. Missing data were handled using pairwise deletion; participants with incomplete responses were excluded only from the specific analyses for which data were missing.

## Results

### Patient characteristics

**Figure 1.**
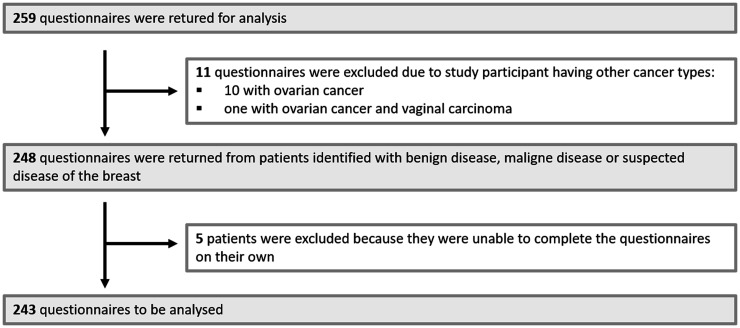
Patient flowchart.

A total of 259 questionnaires were collected for analysis. Eleven questionnaires were excluded due to patients having a diagnosis unrelated to the breast. Five patients were unable to complete the questionnaire themselves and were also excluded. Therefore, the 243 questionnaires remained for analysis (Figure 1). Since the number of distributed questionnaires was not systematically documented, the response rate remains unknown. The majority of patients (94.5%) owned a smartphone at the time of completing the questionnaire (Table 1). The mean age of the study participants was 54.2 (±13.9) years. Most patients were native German speakers (86.4%), and 60.5% had an educational level equal to or higher than a high school diploma (Table 1). The main reasons for presentation included systemic breast cancer therapy (32.6%), follow-up care (23.6%), or mammography/breast ultrasound examination (21.9%; Table 2). Most participants (73.2%) had visited or stayed at the University Hospital Erlangen for 10 days or more (Table 2). However, nearly 26% of patients reported feeling insecure—either “rather insecure” (23%) or “very insecure” (2.6%)—about the information they received during consultations (Table 2).

**Table 1.** Sociodemographic patient characteristics.

Variables	*n*	%
Age		
Mean (SD)	54.2 (13.9)	
Median (min-max)	56 (19–82)	
Age group		
≤60	152	62.6
>60	91	37.4
First language		
German	210	86.4
Other	33	13.6
Educational status		
<high school diploma	96	39.5
≥high school diploma	147	60.5
Previous clinic experience		
Yes	199	84.3
No	37	15.7
Missing	7	
Previous contact with in-person patient navigator		
Yes	33	13.8
No	206	86.2
Missing	4	
Current smartphone ownership		
Yes	225	94.5
No	13	5.5
Missing	5	

Values are expressed as numbers or percentages with respect to total sample size (*n* = 243).

*n*: number; SD: standard deviation; min: minimum; max: maximum.

**Table 2.** Reason for presentation, health status and experience with healthcare system.

Variables	*n*	%
Main reason for presentation at the hospital		
Discussion of findings	30	12.4
Mammography / breast ultrasound examination	53	21.9
Preoperative information	5	2.1
Systemic breast cancer therapy	79	32.6
Follow-up care	57	23.6
Other	18	7.4
Missing	1	
Disease		
Suspicious disease of the breast	38	17.3
Benign disease of the breast	10	4.5
Breast cancer	141	64.1
Other	31	14.1
Missing	21	
Number of days at University Hospital Erlangen		
First contact	19	8.2
2–10 days	43	18.6
>10 days	169	73.2
Missing	12	
Confidence after obtaining new medical information during consultations		
Very secure	31	13.2
Rather secure	99	42.3
Neutral	43	18.4
Rather unsecure	55	23.5
Very unsecure	6	2.6
Missing	9	

Values are expressed as numbers or percentages with respect to total sample size (*n* = 243).

*n*: number.

### Assessment of previous experience with in-person patient navigators and provided support

An evaluation of prior experience revealed that 40.3% of respondents had already heard about patient navigators at the University Hospital Erlangen (Table 3). In total, 33 patients (13.8%) previously had contact with them. Among those, more than 90% rated the support positively, 60.6% “very helpful and supportive,” and 30.3% “helpful and supportive” (Table 3, Supplementary Figure S1). The most commonly received support categories included help with treatment planning and coordination (42.4%), provision of information (39.4%), local orientation within the hospital (36.4%), and emotional support and guidance (30.3%) (Table 3). Further questions regarding preferred media for receiving information revealed that patients most frequently favored text-based materials (55.6%; e.g., written texts and brochures), followed by audiovisual content (42.8%; e.g., videos, animations). Interactive tools were identified by 26.7% of patients as a preferred medium for a future digital patient navigator app (Table 3).

**Table 3.** Experience with in-person patient navigators and expectations of a digital patient navigator.

	*n*	%
Have you ever heard from the patient navigators at the University Hospital Erlangen?		
Yes	96	40.3
No	142	59.7
Missing	5	
Have you already had contact with patient navigators at the University Hospital Erlangen?		
Yes	33	13.8
No	206	86.2
Missing	4	
If you have already had contact with a patient navigator, how would you rate the support you received?		
Very helpful and supportive	20	60.6
Helpful and supportive	10	30.3
Neutral	1	3.0
Not very helpful	1	3.0
Not helpful at all	0	0
What kind of support have you received from a patient navigator?^1^		
Scheduling and coordination	14	42.4
Provision of information and clarification	13	39.4
Assistance with administrative tasks	5	15.2
Emotional support and guidance	10	30.3
Local orientation at the University Hospital	12	36.4
Other	4	12.1
Missing	2	
What media would you most like to have in a digital patient navigator app to educate yourself about procedures and treatments?^1^		
Text-based information (infotexts, brochures, etc.)	135	55.6
Audiovisual content (videos, animations, etc.)	104	42.8
Interactive tools (simulations, virtual tours, etc.)	65	26.7
Other	2	0.8
Missing	50	

1 Multiple answers were allowed; n: number.

### Identification of basic key features for a future digital patient navigator app

To determine which app features were considered most important and helpful, we evaluated questionnaire responses on various potential functions. The following five features were most frequently rated as “very important”: (1) direct communication with the attending physician (61%), (2) a personalized treatment plan including information on appointments, medications and therapies (50%), (3) real-time notifications for upcoming appointments and medications (48%), (4) digital information and mediation activities for various support services—such as social services, psycho-oncology, nutritional counseling, complementary medicine, self-help groups, and sports (32%), and (5) 3D guidance at the University Hospital Erlangen (31%).

**Figure 2.**
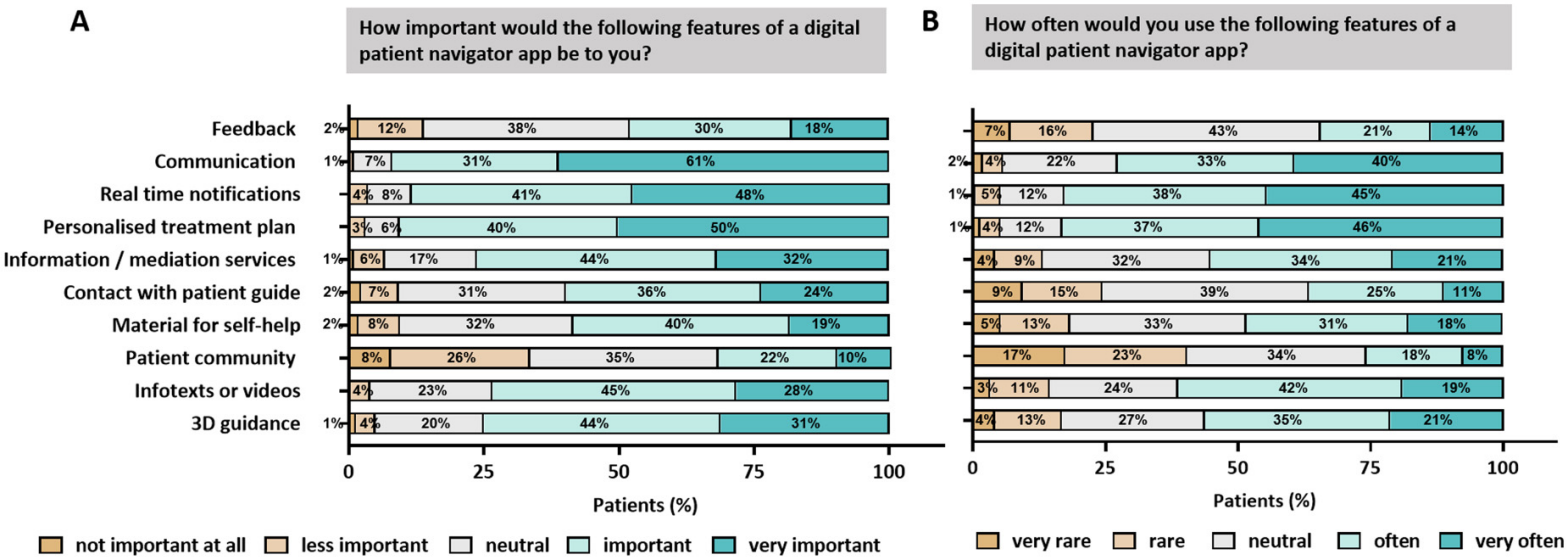
Identification of basic key tools for patient navigator app: Most important A. and most frequently B. used features analyzed on a 5-point Likert scale.

Features considered less important included contact with other patients to exchange information (patient community) (10%), feedback (18%), and access to self-help materials (19%; Figure 2A).

The most frequently used features—rated as “very often”—were as follows: (1) personalized treatment plan (46%), (2) real-time notifications for appointments and medications (45%), (3) direct communication with the attending physician (40%), (4) digital information and mediation activities (21%), and (5) 3D guidance at the University Hospital Erlangen (21%, Figure 2B).

### Patient characteristics with significant influence on the importance of basic key app features

Associations between patient characteristics and the reported importance of each of the five identified basic key features (personalized treatment plan, real-time notifications, communication feature, information and mediation services, and 3D guidance) were examined using descriptive analysis. The parameters and tested groups were as follows: (1) age (≤60 years vs. >60 years),^29^ (2) first language (German vs. other languages), (3) educational status (<high school diploma vs. ≥high school diploma), (4) previous clinic experience (yes vs. no), and (5) previous contact with in-person patient navigators (yes vs. no). Significant differences were mainly observed for two features: personalized treatment plan and real-time notifications. For the personalized treatment plan, the analysis revealed a median (Md) score of 5.00 with an interquartile range (IQR) of 2.00–5.00 for the age group ≤60 years and a score of 4.00 (IQR 2.00–5.00) for the group >60 years, resulting in a statistically significant difference (*p* < 0.001, Table 4). For real-time notifications, the age group ≤60 years reported a higher Md score of 5.00 (IQR 2.00–5.00), compared to 4.00 (IQR 1.00–5.00) in the group >60 years (*p* = 0.036). Additionally, participants with a lower educational status had a higher Md score for real-time notifications (5.0, IQR 2.00–5.00 vs. 4.00, IQR 2.00–5.00, *p* = 0.003; Table 4). No significant influence was found regarding the importance of these two app features based on prior contact with in-person patient navigators or previous clinic experience. Furthermore, there were no statistically significant associations between any of the tested characteristics—age group, first language, educational status, clinical experience, or previous contact—and the app features information/mediation services and 3D guidance (Table 4).

**Table 4.** Assessment of importance of basic app features in relation to various patient parameters.

		Personalized treatment plan (Likert 1–5)			Real time Notifications (Likert 1–5)			Communication (Likert 1–5)			Information and Mediation (Likert 1–5)			3D guidance (Likert 1–5)		
Demographic Variable	Category	*n*	Median (IQR)	*p* value	*n*	Median (IQR)	*p* value	*n*	Median (IQR)	*p* value	*n*	Median (IQR)	*p* value	*n*	Median (IQR)	*p* value
Age (years)	≤60	143	5.00 (2.00–5.00)	<0.001	145	5.00 (2.00–5.00)	0.036	145	5.00 (2.00–5.00)	0.351	145	4.00 (2.00–5.00)	0.606	143	4.00 (1.00–5.00)	0.212
	>60	80	4.00 (2.00–5.00)		80	4.00 (2.00–5.00)		79	5.00 (2.00–5.00)		81	4.00 (2.00–5.00)		81	4.00 (1.00–5.00)	
Native Language	German	204	4.00 (2.00–5.00)	0.615	207	4.00 (2.00–5.00)	0.053	205	5.00 (2.00–5.00)	0.041	207	4.00 (2.00–5.00)	0.284	205	4.00 (1.00–5.00)	0.822
	Other	19	5.00 (2.00–5.00)		18	5.00 (4.00–5.00)		19	5.00 (3.00–5.00)		19	5.00 (2.00–5.00)		19	4.00 (2.00–5.00)	
Educational status	<High school diploma	136	5.00 (2.00–5.00)	0.125	136	5.00 (2.00–5.00)	0.003	136	5.00 (2.00–5.00)	0.654	139	4.00 (2.00–5.00)	0.838	137	4.00 (1.00–5.00)	0.133
	≥High school diploma	87	4.00 (2.00–5.00)		89	4.00 (2.00–5.00)		88	5.00 (2.00–5.00)		87	4.00 (2.00–5.00)		87	4.00 (1.00–5.00)	
Previous clinic experience	Yes	183	5.00 (2.00–5.00)	0.485	185	4.00 (2.00–5.00)	0.259	184	5.00 (2.00–5.00)	0.189	186	4.00 (2.00–5.00)	0.903	185	4.00 (1.00–5.00)	0.185
	No	35	4.00 (2.00–5.00)		35	5.00 (2.00–5.00)		35	5.00 (3.00–5.00)		35	4.00 (2.00–5.00)		34	4.00 (3.00–5.00)	
Previous contact with patient navigators	Yes	30	5.00 (3.00–5.00)	0.634	30	4.00 (3.00–5.00)	0.980	29	5.00 (3.00–5.00)	0.781	30	4.00 (3.00–5.00)	0.941	30	4.00 (3.00–5.00)	0.893
	No	191	4.00 (2.00–5.00)		193	4.00 (2.00–5.00)		193	5.00 (2.00–5.00)		194	4.00 (2.00–5.00)		192	4.00 (1.00–5.00)	

IQR: interquartile range; *n*: number; Likert scale options: 1 = not important at all, 2 = less important, 3 = neutral, 4 = important, 5 = very important.

### Patient characteristics with significant influence on the assessment of frequency of use of basic key app features

In addition to importance ratings, the anticipated frequency of use of the five key app features was analyzed (Table 5). For the personalized treatment plan, statistically significant differences in the frequency of use were observed: participants in the younger age group reported higher use (Md = 5.00, IQR 1.00–5.00) compared to the group >60 years (Md = 4.00, IQR 1.00–5.00, *p* < 0.001), and participants with lower educational levels reported higher use (Md = 5.00, IQR 2.00–5.00) compared to the group with higher educational levels (Md = 4.00, IQR 1.00–5.00, *p* = 0.025, Table 5). Statistically significant differences were also observed in the expected use of the real-time notification feature: participants ≤60 years reported higher use (Md = 5.00, IQR 2.00–5.00) compared to older individuals (Md = 4.00, IQR 1.00–5.00, *p* = 0.009). Likewise, participants with lower educational levels reported higher use (Md = 4.50, IQR 2.00–5.00) compared to those with higher educational levels (Md = 4.00, IQR 1.00–5.00, *p* = 0.028).

**Table 5.** Assessment of frequency of use of basic app features in relation to various patient parameters.

		Personalized treatment plan (Likert 1–5)			Real time Notifications (Likert 1–5)			Communication (Likert 1–5)			Information and Mediation (Likert 1–5)			3D guidance (Likert 1–5)		
Demographic Variable	Category	*n*	Median (IQR)	*p* value	*n*	Median (IQR)	*p* value	*n*	Median (IQR)	*p* value	*n*	Median (IQR)	*p* value	*n*	Median (IQR)	*p* value
Age (years)	≤60	138	5.00 (1.00–5.00)	<0.001	137	5.00 (2.00–5.00)	0.009	137	4.00 (1.00–5.00)	0.613	138	4.00 (1.00–5.00)	0.275	138	4.00 (1.00–5.00)	0.209
	>60	75	4.00 (1.00–5.00)		78	4.00 (1.00–5.00)		76	4.00 (1.00–5.00)		77	4.00 (1.00–5.00)		77	4.00 (1.00–5.00)	
First Language	German	197	4.00 (1.00–5.00)	0.061	200	4.00 (1.00–5.00)	0.080	198	4.00 (1.00–5.00)	0.055	199	4.00 (1.00–5.00)	0.306	200	4.00 (1.00–5.00)	0.208
	Other	16	5.00 (2.00–5.00)		15	5.00 (2.00–5.00)		15	5.00 (2.00–5.00)		16	4.00 (2.00–5.00)		15	4.00 (1.00–5.00)	
Educational status	<High school diploma	131	5.00 (2.00–5.00)	0.025	132	4.50 (2.00–5.00)	0.028	131	4.00 (1.00–5.00)	0.735	132	4.00 (1.00–5.00)	0.594	132	4.00 (1.00–5.00)	0.869
	≥High school diploma	82	4.00 (1.00–5.00)		83	4.00 (1.00–5.00)		82	4.00 (1.00–5.00)		83	4.00 (1.00–5.00)		83	4.00 (1.00–5.00)	
Previous clinic experience	Yes	178	4.00 (1.00–5.00)	0.144	180	4.00 (2.00–5.00)	0.383	177	4.00 (1.00–5.00)	0.460	179	4.00 (1.00–5.00)	0.619	179	4.00 (1.00–5.00)	0.679
	No	32	5.00 (1.00–5.00)		32	5.00 (1.00–5.00)		33	4.00 (1.00–5.00)		33	4.00 (1.00–5.00)		33	4.00 (1.00–5.00)	
Previous contact with patient navigators	Yes	27	4.00 (1.00–5.00)	0.575	29	4.00 (1.00–5.00)	0.141	29	4.00 (1.00–5.00)	0.235	28	4.00 (1.00–5.00)	0.692	28	4.00 (1.00–5.00)	0.992
	No	185	4.00 (1.00–5.00)		185	4.00 (2.00–5.00)		183	4.00 (1.00–5.00)		186	4.00 (1.00–5.00)		186	4.00 (1.00–5.00)	

IQR: interquartile range; Likert scale options: 1 = very rarely, 2 = rarely, 3 = neutral, 4 = frequent, 5 = very frequent.

For all other app features, no statistically significant differences were observed between the tested groups regarding age, first language, educational status, previous clinic experience, or prior contact with in-person patient navigators (Table 5).

### Suggestions and ideas for additional app features

Respondents were additionally invited to provide ideas and express their preferences for app features in a free-text field. Suggestions were sorted into the following six categories: (1) findings, reports, and therapy; (2) appointment optimization and waiting time reduction; (3) advice and assistance with adverse reactions and patient motivation; (4) parking; (5) contact with medical professionals; and (6) general suggestions (Table 6). The majority of suggestions fell within the category “findings, reports and therapy” (32.1%). In detail, patients suggested saving answers to relevant and recurring questions in the app or recommended a traffic light system with green (no appointment needed), yellow (appointment needed), and red color (appointment urgently needed) to provide a quick overview of current results. A total of 21.4% of the respondents made suggestions related to “appointment optimization and waiting time reduction,” such as recommendations for cafés and canteens or an option for notifications about expected waiting time. Additionally, patients proposed motivational content, a patient community feature, and information about activities to minimize adverse effects of, for example, from chemotherapy, as shown in the category “advice and help with adverse reactions and patient motivation” (17.9%). Other key areas of interest included “parking” (10.7%) as well as “contact with medical professionals” (10.7%). General suggestions included the provision of free Wi-Fi and the availability of an English app version (Table 6).

**Table 6.** Suggestions (*n* = 28) and ideas for additional app features showing categories and exemplary answers.

Category classification	*n*	%
Findings, reports and therapy	9	32.1
* … providing of blood values, reports, results (MRT, CT) and findings in app, also from other clinic departments*		
* … traffic light system for radiological findings: red (appointment urgently needed), yellow (appointment needed) and green (no appointment needed)*		
* … saving of questionnaires with the most important informations (allergies, medication, age, weight, height, medical history…)*		
* … possibility of digital transfer of referral slips directly to hospital*		
* … providing of specific treatment suggestions before consultation of physician*		
Appointment optimizing and waiting time reduction	6	21.4
* … possibility of combining appointments via app, especially for external patients*		
* … notification of expected waiting time and notification if an appointment will be postponed*		
* … cafes and canteens*		
* … shortening the registration process by ability of previously filling out forms*		
Advice and help with adverse reactions and patient motivation	5	17.9
* … information about sports or activities to minimize adverse effects (e.g., of chemotherapy)*		
* … motivational content such as: “You can do it!” … questions about daily form, sleep etc. “How are you feeling today?,” “Do you have pain?”*		
* … inclusion of patient community with tips and tricks for hair, make-up, hats, furry hands and feet*		
* … patient diary for documentation of pain, intolerances, questions and notes*		
Parking	3	10.7
… *information about parking facilities*		
Contact with medical professionals	3	10.7
* … brief profiles of the attending physician and nurses*		
* … possibility of contacting the attending physician*		
* … providing a telephone number for questions*		
General suggestions	2	7.1
* … app should also be available in English*		
* … free WiFi to use the app*		

*n*: number.

### Further patient feedback and wishes

A total of 14 patients provided further feedback and expressed their preferences and expectations for a future digital navigator app. The received suggestions (*n* = 15) were grouped into four categories: (1) general positive feedback, (2) further suggestions regarding waiting time, contact with physicians, local orientation, and findings, (3) considerations for older people and those without a mobile phone, and (4) negative feedback (Table 7). The majority of patients made further suggestions related to the category “waiting time, contact with physicians, local orientation, and findings” (40%), recommending the option to contact a physician as an important app function, as well as reduced waiting times and access to individual results (Table 7); 13.3% offered general positive feedback and expressed appreciation for the app concept. Only four patients (26.7%) were critical of the app, indicating a lack of interest in using it. They also stated that digital apps cannot fully substitute for interpersonal contact. Another point raised was the app's usability for older individuals and those without access to smartphones (20.0%). In this context, patients proposed a straightforward design and interface, along with the option to borrow smartphones (Table 7).

**Table 7.** Further feedback and suggestions (*n* = 15), showing categories and exemplary answers.

Category classification	*n*	%
General positive feedback	2	13.3
* … very good idea, application should be as simple as possible*		
* … very good initiative, app is highly expected*		
Further suggestions regarding waiting time, contact with physicians, local orientation and findings	6	40.0
* … good WiFi signal, app should be compatible with all smartphones*		
* … possibility of contacting the attending physician in writing*		
* … bringing together different aspects of an underlying disease*		
* … shortening of waiting time by presenting results in app*		
* … for local orientation*		
Considerations for older people and those without smartphone	3	20.0
* … alternatives for people without a mobile phone, rental devices*		
* … simple design and handling of app for seniors*		
Negative feedback	4	26.7
… *do not want to use any digital app*		
… *digital platforms cannot replace human to human contact*		

*n*: number.

## Discussion

The main aim of this study was to query patients about their preferences regarding the content and features of a digital patient navigator app. To the best of our knowledge, this is the first study to explore patients’ preferences and integrate descriptive results at an early stage to involve patients in shaping the app according to their wishes.

There are already many health applications on the market that are subject to minimal regulation. Development is largely driven by industry, with the goal of reaching a broad sales market as easily as possible. However, many apps are rarely used, tend to be superficial, and offer little added value for patients.^27^ To ensure regular use, a user-centered app design—based on the abilities of the users rather than those of medical staff or developers—is essential^30^ to minimize the risk of creating an unusable app.^31^ Therefore, user involvement is a crucial part of software development processes. A lack of user involvement can result in a poorly designed solution, reduce the chances of successful implementation, and create conflicts with users’ needs.^25,32^ Patient participation has also been shown to enable the identification of complications unrecognized by researchers and elucidate the specific interests of the target population.^33,34^ In this context, content validity is of particular relevance. Content validity addresses whether a tool adequately captures the intended construct, is appropriate and meaningful for the target user group, and fits the context of use in app-based interventions.^35,36^ A five-step process for the development of eHealth applications has been defined: (1) establish content; (2) establish eHealth literacy; (3) establish technology delivery; (4) establish expert usability; and (5) establish participant usability.^37^ By conducting a user-centered survey, we focused on the first step of eHealth application development: content. Within this process, user engagement can help to tailor the digital tool to the users’ needs and provide content validity for the final product.^38^ By assessing user needs and preferences at an early stage, this study helps to ensure that future app development is aligned with patient expectations.

In 2022, the first Patient Navigator Day was held as a joint event. More than 45 patient navigator projects across Germany are currently ongoing, supporting over 75,000 people with complex life situations and a variety of medical conditions.^39^ The potential of in-person patient navigators is substantial, and they are important for advancing the healthcare system in terms of sustainable, quality-assured, and patient-oriented care.^40^ The acceptance and value of in-person patient navigators were also confirmed in this study: 13.6% of our participants had contact with an in-person patient navigator, and over 90% of them rated the support as helpful or very helpful and supportive. Findings from another study conducted in Germany showed that most people predominantly expect in-person patient navigators to provide administrative assistance, regardless of their socioeconomic or health status.^41^ Interestingly, the three most frequently used types of support can be handled effectively by a digital navigator: planning and coordination of treatment, provision of information, and local orientation at the hospital. Such features could easily be integrated into a patient navigator app, helping patients manage their appointments on a routine basis.

In this study, we attempted to define the role of a digital patient navigator by examining the experiences and expectations of patients at a major tumor center. Although many patients were familiar with the university hospital, a quarter of them reported being unsure about the information provided during (Table 2). Another study has shown that a third of patients misunderstand the information they receive.^42^ Existing patient information materials, which are intended to supplement discussions during consultations, are often not presented in a format that is clear, understandable, or helpful. A digital patient navigator could, therefore, be particularly valuable for these patients, supporting them throughout the dynamic treatment process. Moreover, many patients today actively seek detailed information about their diagnosis and treatment options, and many prefer to participate in medical decision-making. This is supported by the growing evidence across various areas of medicine that shows the benefit of involving patients in decision-making and the improved quality of life through personalized mHealth interventions.^43^

In addressing our primary objective of assessing preferences for the content and functionality of a digital patient navigator, we found that the majority of respondents had a positive attitude toward a digital patient navigator app and preferred personalized features (e.g., an individual treatment plan or real-time notifications for upcoming treatments). The assessment of the anticipated frequency of use supported these findings.

Besides analyzing the preferences and frequency of use of basic app features, we further investigated how different patient characteristics correlate with preferences for specific app functions. Patients younger than 60 years considered a personalized treatment plan and real-time notifications for appointments, examinations, and other important results significantly more important than older patients. This aligns with the observation that younger individuals are generally more familiar with internet-based services and, therefore, more likely to embrace mobile technology. It has been shown that age and education are major factors in the adoption of digital health apps among those seeking cancer information.^44^ Regarding educational level, patients with lower education rated the personalized treatment plan as more important and indicated they would potentially use this feature more often than those with higher education. This group might benefit considerably from the patient navigator app, particularly in terms of support with appointments, medication, and therapies. Patients whose first language is not German rated the real-time notifications feature as more important than native German speakers and tended to anticipate more frequent use of features such as the personalized treatment plan, real-time notifications, and communication tools.

Although subgroup analyses showed statistically significant differences in the perceived importance and expected frequency of use for certain features, these differences should be interpreted cautiously. Median values were consistently high (4.0 or 5.0) across all subgroups, indicating that these functions are considered important by most patients—regardless of age, education, or language background. The differences observed are likely more reflective of subtle variations in digital familiarity or expectations than of generalizable differences in needs. Nevertheless, these insights can inform practical design choices—for example, simplifying content, offering multilingual access, or adapting the interface to better support older patients or those with lower health or digital literacy.

It would be a significant advantage if the app supported multiple languages, allowing patients to better understand all the information provided. In our study, approximately one in six respondents reported a native language other than German. At the end of 2022, around 13.38 million foreigners and 24 million people with a migration background were living in Germany.^45^ The integration of data and information in different languages could be a key benefit of a digital patient navigator, helping to overcome language-related barriers between patients and medical professionals. A study investigating the differences of interpreters and bilingual patient navigators showed that a bilingual patient navigator was particularly beneficial for building trust, restate physician speech into plain language, and to teach basic skills for medical appointments.^46^ In addition, several studies showed that interpretation apps can help to reduce barriers related to linguistic diversity.^47^ Whether a digital, multilingual patient navigator could achieve similar effects remains to be further explored in future research.

Our data suggest that certain features—such as a personalized treatment plan, real-time notifications, and direct communication with physicians—are considered highly important and likely to be used frequently by patients. These are precisely the functions that currently consume significant staff time during patient visits. Therefore, while our study does not provide direct evidence for workflow optimization or improved health outcomes, it does highlight specific areas where digital support is both desired and likely to be adopted. These patient preferences should inform the next steps in prototype development and future evaluation studies, which can formally assess the impact on clinical efficiency, staff workload, and patient outcomes.

Workflow organization through digital tools might become increasingly important in the future. The current German hospital reform, initiated in 2023 by the Federal Ministry of Health, aims to restructure inpatient care through the introduction of service groups, minimum quality criteria, and a shift toward needs-based hospital planning and cross-sector care facilities.^48^ As part of this reform, cancer patients will be treated almost exclusively in large centers, which will initially present significant organizational challenges. This reflects the increasing complexity of oncology, as seen in breast cancer treatment, where multidisciplinary coordination is essential for informed decision-making.^49^ Thus, digital tools like patient navigator apps will become more and more important to help patients navigate through large facilities and complex treatment structures. Patients are often uncertain when confronted with many different specialists, making it clear that meaningful support—independent of a single physician—is essential. A personalized schedule provided by the digital navigator, containing all necessary information, could optimize the use of urgently needed professional human resources. With a plan that patients can understand and follow, we hope that adherence will improve and be more effective compared to the currently existing navigation systems.

**Figure 3.**
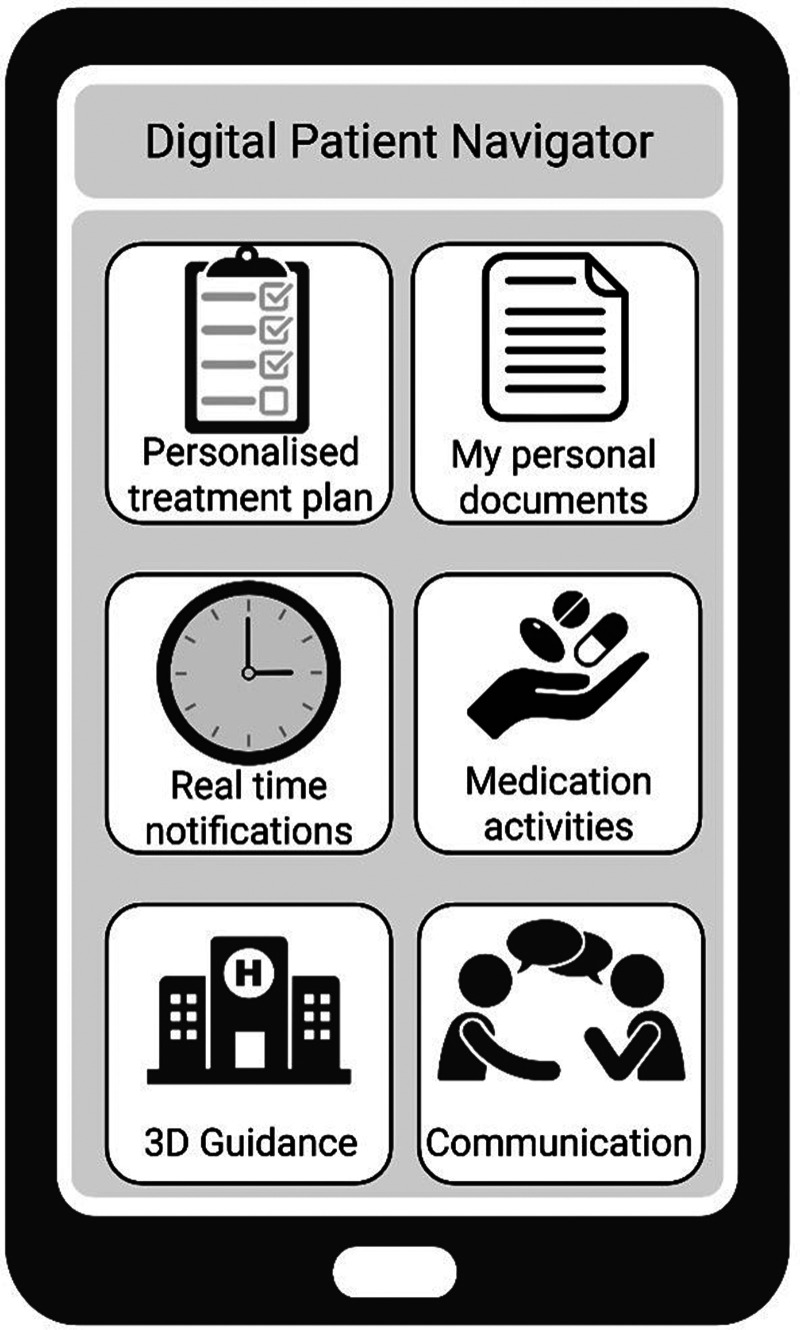
Vision of a future digital patient navigator app: App functions are based on the questionnaire analysis and are adapted to patients’ preferences. Personalized treatment plan: informations about appointments, medications and therapies; My personal documents: blood values, reports, results and saving of important questionnaires; Real-time notifications: for upcoming appointments and examinations; Mediation activities and digital information: social services, psycho-oncologists, nutritional counseling, complementary medicine, self-help groups and sports, tips and tricks for hair and make-up; 3D Guidance: informations about parking facilities, cafes and canteens; Communication: direct possibility of communication with attending physician. Created in BioRender.

An app developed based on patients’ needs should reflect their perspectives on patient-centered care. The presented results of our survey provide a good foundation for designing a patient navigator app prototype that aligns with these preferences. If defining a prototype user interface, it should include the following core app features identified as most important by patients (Figure 3): (1) personalized treatment plan: information about appointments, medications and therapies; (2) my personal documents: blood values, reports, and storage of important questionnaires; (3) real-time notifications: for upcoming appointments and examinations; (4) mediation activities and digital information: social services, psycho-oncologists, nutritional counseling, complementary medicine, self-help groups and sports, tips and tricks for hair and make-up; (5) 3D guidance: navigation through the clinic and information about parking facilities, cafés, and canteens; and (6) communication: direct possibility of communication with the attending physician.

Real-time notifications for upcoming appointments and medications, digital information, and mediation activities for various support services, and 3D hospital guidance are all tools that can be integrated into a digital patient navigator. It is quite clear that such a product should cover these areas. An initial test version and a technical consultation have shown that the desired features are, for the most part, technically feasible. For future implementation and broader application, adjustments to data protection regulations in Germany will be necessary, and the integration into the European data space—such as the Gaia-X project—could be beneficial to support interoperable depoyment.^50,51^ Further studies should now evaluate the practical impact and usability of these tools in real-world clinical settings.

## Limitations

Our study has several limitations that should be considered when interpreting the results:

First, the study was conducted using a German questionnaire, which may have disadvantaged participants with limited German language skills. Although we excluded patients who were unable to understand the German language, language barriers may have led to the underrepresentation of some groups.

Second, the questionnaire was developed specifically for this study. Its content was based on literature, clinical experience, and both expert and patient input, which helped ensure relevance and clarity. While this approach supports content validity, no formal psychometric validation was performed.

Third, the study focused exclusively on the patient's perspective. While this was intentional for this stage of development, the involvement of other stakeholders—such as physicians and nurses—in the future will also be important and may lead to further improvements. Their perspectives may differ from those of patients and could influence priorities.

Fourth, the study was conducted as a single-center study focusing on patients with breast disease; therefore, the results are not generalizable to other disease areas. Further research may be needed to develop a digital patient navigation system with broader applicability.

Fifth, the cross-sectional design of our study captured patient preferences only at the time of questionnaire completion. These preferences may change over the course of treatment or with growing experience in the clinical setting. However, the vast majority (84.3%) had previous experience with outpatient or inpatient care, so it can be assumed that insights from earlier encounters may have influenced their responses.

Sixth, the total number of distributed questionnaires was not documented; as a result, the response rate remains unknown, and the non-response bias could not be assessed. It is possible that patients who were more digitally engaged, had more time during their visit, or had a stronger interest in the topic were more likely to participate. As such, the results may overrepresent the views of more motivated or digitally literate individuals and may not fully reflect the preferences of patients with limited interest in digital health tools or lower digital literacy.

Seventh, we performed multiple group comparisons using Mann–Whitney *U* tests without correction for multiple testing. Given the exploratory nature of the study, this approach was chosen to avoid missing relevant associations; however, the increased risk of Type I errors must be acknowledged. Reported *p*-values should therefore be interpreted with caution, and findings confirmed in future studies.

Lastly, before building the final app, practical requirements need to be clarified. For example, direct communication with physicians might not be feasible, as it would require additional time and personnel resources. The technical and organizational feasibility of such features should therefore be explored in subsequent phases of development.

## Conclusions

Our study represents the first step in the development of a digital patient navigator app and focuses on identifying patients’ needs and preferences during the initial stages of app development. Key features identified for the future app include personalized and interactive elements. The app should have a user-friendly interface and be “as simple as possible.” However, variations in expectations regarding the personalized treatment plan and real-time notifications were observed based on age, educational status, and first language. It is important to consider these differences in the further development of patient navigation models, offering greater flexibility and adaptability for different patient groups. Continued development of the healthcare system is essential to optimize patient-centered and population-oriented support. By utilizing available technologies, patient satisfaction and treatment quality can be improved, benefiting patients while also reducing the burden on medical professionals.

## Supplemental Material

sj-docx-1-dhj-10.1177_20552076251387746 - Supplemental material for Cross-sectional study on user requirements for developing a digital patient navigator app Supplemental material, sj-docx-1-dhj-10.1177_20552076251387746 for Cross-sectional study on user requirements for developing a digital patient navigator app by Katharina Seitz, Manuela Langbein, Chloë Goossens, Stefanie Altmannshofer, Peter A. Fasching, Carolin C. Hack, Felix Heindl, Matthias W. Beckmann, Julius Emons and Hanna Huebner in DIGITAL HEALTH

sj-docx-2-dhj-10.1177_20552076251387746 - Supplemental material for Cross-sectional study on user requirements for developing a digital patient navigator app Supplemental material, sj-docx-2-dhj-10.1177_20552076251387746 for Cross-sectional study on user requirements for developing a digital patient navigator app by Katharina Seitz, Manuela Langbein, Chloë Goossens, Stefanie Altmannshofer, Peter A. Fasching, Carolin C. Hack, Felix Heindl, Matthias W. Beckmann, Julius Emons and Hanna Huebner in DIGITAL HEALTH
