# Supplementary material for: Cross-sectional study on user requirements for developing a digital patient navigator app
Source: Digit Health. 2025 Oct 28;11:20552076251387746. doi: 10.1177/20552076251387746 (PMC12576095; doi:10.1177/20552076251387746)

Figure S 1: Assessment of previous experience with in-person patient navigators (n=33) (A) and type of support provided by in-person patient navigators (B, multiple answers possible).


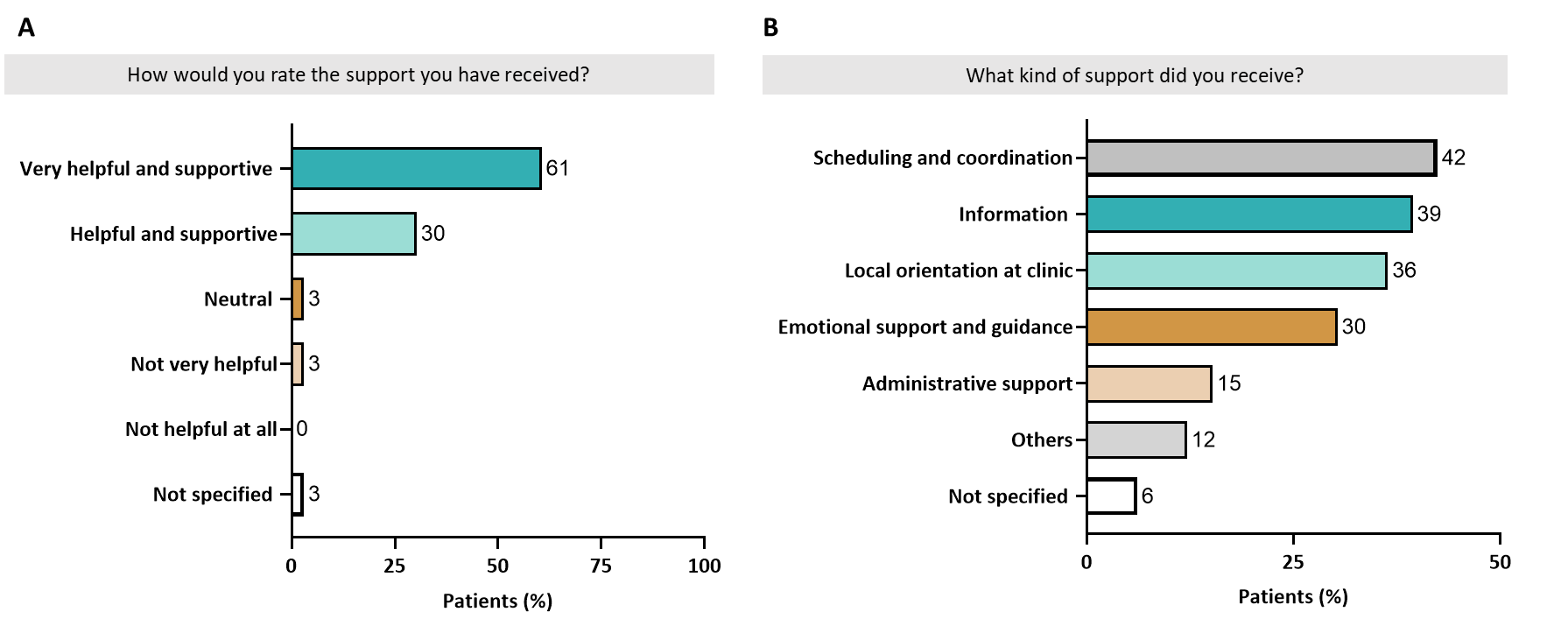

Supplement: sj-docx-1-dhj-10.1177_20552076251387746 - Supplemental material for Cross-sectional study on user requirements for developing a digital patient navigator app [file sj-docx-1-dhj-10.1177_20552076251387746.docx]
