# Supplementary material for: Cross-sectional study on user requirements for developing a digital patient navigator app
Source: Digit Health. 2025 Oct 28;11:20552076251387746. doi: 10.1177/20552076251387746 (PMC12576095; doi:10.1177/20552076251387746)
Supplement: sj-docx-2-dhj-10.1177_20552076251387746 - Supplemental material for Cross-sectional study on user requirements for developing a digital patient navigator app [file sj-docx-2-dhj-10.1177_20552076251387746.docx]

Supplementary File 1: Translated version of the questionnaire

**1. Demographic information**

| **1.1 How old are you** **(in years)?** | _______ Years | |
| --- | --- | --- |
| **1.2 Which of the following languages are your native languages? (multiple answers possible)** | ☐ German ☐ English ☐ Turkish ☐ Ukrainian  ☐ Russian ☐ Spanish ☐ Italian ☐ French ☐ Arabic  ☐ Other (please specify): ________________________________ | |
| **1.3 What is your highest level of education?** | ☐ None  ☐ Secondary school certificate  ☐ Vocational training  ☐ Master's degree | ☐ Secondary school leaving certificate  ☐ A-levels  ☐ Bachelor's degree  ☐ Doctorate |
| **1.4 What is your current employment status?** | ☐ Full-time employment  ☐ Self-employed / freelance  ☐ Retired  ☐ Other (please specify): | ☐ Part-time employment  ☐ Not gainfully employed / unemployed ☐ Student / pupil  ________________________________ |
| **1.5 To which professional group would you most likely assign yourself?** | ☐ Healthcare (doctor, nurse, therapist, etc.)  ☐Administration / office work  ☐ Technology / IT  ☐ Services / sales | ☐ Education / teaching  ☐ Craft / production  ☐ Student / pupil  ☐ Other (please specify): _________________________________ |
| **1.6 Do you currently own a smartphone (cell phone)?** | ☐ Yes ☐ No | |

**2. Reason for presentation, health status and experience with the healthcare system**

| **2.1 What is the main reason for your hospital attendance today?** | ☐ Discussion of findings  ☐ Mammography / breast ultrasound  ☐ Operation (information)  ☐ Systemic therapy for breast cancer (chemotherapy; targeted therapy  e.g. antibody therapy, immunotherapy; radiotherapy)  ☐ Follow-up care with endocrine therapy if necessary therapy e.g. letrozole,  tamoxifen etc.  ☐ Other (please specify): _________________________________ |
| --- | --- |
| **2.2 What illness are you attending the clinic for today?** | ☐ Suspected disease of the breast  ☐ Benign disease of the breast  ☐ Breast cancer limited to the breast and possibly lymph nodes (not  metastasized)  ☐ Metastatic breast cancer  ☐ Other (please specify): _________________________________ |
| **2.3 Have you already received one or more of the following treatments in the past?** **(multiple answers possible)** | ☐ Surgery  ☐ Chemotherapy  ☐ Radiation therapy  ☐ Targeted therapy, e.g. antibody therapy or immunotherapy  ☐ Anti-hormone therapy  ☐ None of the above |
| **2.4 Are you currently planning one or more of the following treatments?****(multiple answers possible)** | ☐ Surgery  ☐ Chemotherapy  ☐ Radiation therapy  ☐ Targeted therapy, e.g. antibody therapy or immunotherapy  ☐ Anti-hormone therapy  ☐ None of the treatments mentioned |
| **2.5 Have you already had experience with clinics, e.g. previous outpatient or inpatient hospital stays?** | ☐ Yes ☐ No |
| **2.8 How many days (including today) did you spend at the University Hospital Erlangen?** | ☐ 1 day (first contact today)  ☐ 2-5 days  ☐ 6-10 days  ☐ > 10 days |
| **2.9 How familiar are you already with the locations and facilities at University Hospital Erlangen?** | ☐ Very familiar  ☐ Familiar  ☐ Neutral  ☐ Not very familiar  ☐ Not familiar at all |
| **2.10 How easily do you generally find your way around new places and buildings?** | ☐ Very light ☐ Rather light ☐ Neutral  ☐ Rather heavy ☐ Very heavy |
| **2.11 How confident do you feel in dealing with new medical information that you receive (e.g. during a doctor's consultation)?** | ☐ Very safe ☐ Rather safe ☐ Neutral  ☐ Rather unsafe ☐ Very unsafe |

1. **Experience with the patient navigators**

The University Hospital Erlangen has central contact persons who accompany you before, during and after your treatment at the hospital, the so-called patient navigators. The patient navigators offer you simple, direct and reliable support throughout the course of your treatment. They provide you with assistance during your therapy and ensure the continuity of your oncological treatment.

| **3.1 Have you ever heard about the patient navigators at University Hospital Erlangen?** | ☐ Yes ☐ No |
| --- | --- |
| **3.2 Have you already had contact with the patient navigators at University Hospital Erlangen?** | ☐ Yes ☐ No |
| Please only complete the following two questions in this section if you have already had contact with one of the patient navigators at University Hospital Erlangen | |
| **3.3 If you have already had contact with a patient navigator, how would you rate the support you received?** | ☐ Very helpful and supportive ☐ Helpful and supportive  ☐ Neutral ☐ Not very helpful ☐ Not helpful at all |
| **3.4 What kind of support have you received from a patient navigator? (multiple answers possible)** | ☐ Scheduling and coordination  ☐ Provision of information and clarification  ☐ Assistance with administrative tasks  ☐ Emotional support and guidance  ☐ Local orientation at the University Hospital  ☐ Other (please specify): _________________________________ |

1. **Expectations of a digital patient navigator**

In the following, we would like to find out which functions of a digital patient navigator would be most important and most helpful for you. Accordingly, please imagine that you have an app (i.e. an application on a cell phone or tablet) that is designed to make your visit to the University Hospital Erlangen easier.

We would like to ask you to help us rate the importance of various functions of a digital patient navigator app. Please put a cross next to the respective importance on a scale from "very important" to "not at all important".

| **How important would the following functions of a digital patient navigator be to you...** | very important | important | neutral | less important | not important at all |
| --- | --- | --- | --- | --- | --- |
| **4.1** Digital **3D guidance** for the University Hospital Erlangen to find your way around better |  |  |  |  |  |
| **4.2** **Infotexts or videos** to find out about upcoming medical procedures or treatment |  |  |  |  |  |
| **4.3** **Patient community** where you can exchange information with other patients |  |  |  |  |  |
| **4.4** Access to **resources and self-help materials** on various conditions and medical procedures |  |  |  |  |  |
| **4.5** Contact possibility with the **in-person patient navigators** |  |  |  |  |  |
| **4.6** **Digital information and mediation services** for various **support services** such as social services, psycho-oncologists, nutritional advice, complementary medicine, self-help groups, sports activities |  |  |  |  |  |
| **4.7 Personalized treatment plan** with information on appointments, medication and therapies |  |  |  |  |  |
| **4.8** **Real time notifications** for appointments, examinations and other important events |  |  |  |  |  |
| **4.9 Direct communication** with the attending physician |  |  |  |  |  |
| **4.10** Opportunity to **provide anonymous feedback** on the quality of care and service |  |  |  |  |  |

Please rate the expected frequency of use of the following functions of a digital patient navigator application on a scale from “very frequent” to “not frequent at all”. Please place a cross next to each frequency.

| **How often would you use the following functions...** | very frequent | frequent | neutral | rarely | very rarely |
| --- | --- | --- | --- | --- | --- |
| **4.11** Digital **3D guidance** for the University Hospital Erlangen to find your way around better |  |  |  |  |  |
| **4.12** **Infotexts or videos** to find out about upcoming medical procedures or treatment |  |  |  |  |  |
| **4.13** **Patient community** where you can exchange information with other patients |  |  |  |  |  |
| **4.14** Access to **resources and self-help materials** on various conditions and medical procedures |  |  |  |  |  |
| **4.15** Contact possibility with the **in-person patient navigators** |  |  |  |  |  |
| **4.16** **Digital information and mediation services** for various **support services** such as social services, psycho-oncologists, nutritional advice, complementary medicine, self-help groups, sports activities |  |  |  |  |  |
| **4.17 Personalized treatment plan** with information on appointments, medication and therapies |  |  |  |  |  |
| **4.18** **Real time notifications** for appointments, examinations and other important events |  |  |  |  |  |
| **4.19 Direct** communication with the attending physician |  |  |  |  |  |
| **4.20** Opportunity to provide **anonymous feedback** on the quality of care and service |  |  |  |  |  |

**4.21 Do you have any suggestions or ideas for additional features that could be integrated into the digital patient navigator app to improve the patient experience?**

________________________________________________________________________________________________________________________________________________________________________________________________________________________________________________________________________________________________________________________________________

**4.22 What media would you most like to have in a digital patient navigator app to educate yourself about procedures and treatments?**

☐ Text-based information (infotexts, brochures, etc.)

☐ Audiovisual content (videos, animations, etc.)

☐ Interactive tools (simulations, virtual tours, etc.)

☐ Other (please specify): ____________________________

| **4.23 If you would like to give us further feedback or requests regarding a digital patient navigator, you are welcome to do so here:** |
| --- |
